# Exposure to Juvenile Stress Induces Epigenetic Alterations in the GABAergic System in Rats

**DOI:** 10.3390/genes14030565

**Published:** 2023-02-23

**Authors:** Gil Ben David, Yam Amir, Kuldeep Tripathi, Lital Sharvit, Amir Benhos, Rachel Anunu, Gal Richter-Levin, Gil Atzmon

**Affiliations:** 1 Sagol Department of Neurobiology, Faculty of Natural Sciences, University of Haifa, 199 Aba Khoushy Ave. Mount Carmel, Haifa 3498838, Israel; 2 Department of Human Biology, Faculty of Natural Sciences, University of Haifa, 199 Aba Khoushy Ave. Mount Carmel, Haifa 3498838, Israel; 3 Department of Psychology, Faculty of Social Sciences, University of Haifa, 199 Aba Khoushy Ave. Mount Carmel, Haifa 3498838, Israel; 4 Integrated Brain and Behavior Research Center (IBBR), University of Haifa, 199 Aba Khoushy Ave. Mount Carmel, Haifa 3498838, Israel

**Keywords:** PTSD, epigenetics, DNA methylation, GAD, hippocampus, amygdala, stress

## Abstract

Epigenetics is a gene–environment interaction mechanism, manifested mostly through changes in regulatory gene expression. Stress is an established environmental factor known to induce epigenetic changes. This study aimed to assess the long-term effect of stress as juveniles, or juvenile and adult stress, on alterations in glutamic acid decarboxylase genes (*GAD65*, *GAD67*). We assessed DNA methylation and RNA expression in four rat groups: (1) control group, (2) juvenile stress group sacrificed two days following stress exposure (JSe) (RNA only), (3) juvenile stress group sacrificed as adults (JS), and (4) juvenile and adult stress group (JS + AS). Three different areas of the brain were examined in each group: the dorsal dentate gyrus (dDG), the dorsal CA1 (dCA1), and the basolateral amygdala (BLA). A significantly low methylation level of *GAD65* in the BLA was observed among the JS group, followed by almost complete recovery among the JS + AS group. However, in dDG, an opposite trend was captured, and higher *GAD65* methylation was found in JS. In addition, RNA levels were found to be decreased in JS compared to JSe and JS + AS. These findings can point to a possible mechanism: while juvenile stress may enhance a better coping strategy with life challenges, additional stress in adulthood may trigger a contradictory response, either beneficial or harmful.

## 1. Introduction

Stress can be defined as a personal response to a stimulus perceived as a mental or physical health threat. The factors affecting stress are varied and include physical, psychological, or social stimuli [1]; these factors are known as stressors. Stress usually fades once stressors’ influence ends, and the body returns to homeostasis. In some circumstances, the stressors’ influence continues, and the coping mechanism fails. This may result in long-term biological, behavioral, and cognitive changes [2], which, in extreme cases, may end with pathological conditions such as post-traumatic stress disorder (PTSD).

Exposure to a stressful event activates the hypothalamic–pituitary–adrenal (HPA) axis, along with several other brain regions, including the hippocampus and the amygdala. Specifically, while the hippocampus regulates this axis through the transmission of inhibitory signals, the amygdala excites it by transmitting disinhibitory signals [3,4].

GABA (γ-aminobutyric acid) is the primary inhibitory neurotransmitter in the brain and a major player in anxiety and mood disorders [5]. GABA is converted from glutamate by the enzyme glutamic acid decarboxylase (GAD). GAD is encoded by two different genes, resulting in the synthesis of two distinct isoforms of the enzyme, *GAD65* and *GAD67*. GAD67, located in the cytoplasm, is constitutively active, allowing a steady basal GABA production. In contrast, GAD65 is found primarily in synaptic vesicles and is transiently active in response to sudden increases in GABA demand to fine-tune GABAergic synaptic function [6,7].

Extracellular cues and intracellular signaling machinery are used in various ways to regulate transcription. The transcriptional control of both *GAD67* and *GAD65* has been linked to glutamate and N-Methyl-D-aspartic acid receptor (NMDAR) signaling. The expression of GADs in the nucleus is directly enhanced by neuronal activity via NMDAR followed by Ca^2+^ influx, whereas *GAD67* is the most prominent [8,9]. In a parallel pathway and due to neuronal activity, brain-derived neurotrophic factor (BDNF) is released from excitatory synapses [10,11]. BDNF binds to the TrkB receptor, activating the RAS-ERK-CREB intracellular signaling pathway. This increases the expression of *GADs*, particularly *GAD65*, in GABAergic neurons [9,12].

Numerous studies have shown alterations of GAD in the amygdala and hippocampus as a result of stress exposure [4,13,14,15,16,17,18,19,20]. GAD genetic variation also has often been used as an indicator of GABAergic-related function. Further, systemic manipulation of GABAergic enzymes is well known to strongly modulate anxiety-like behaviors; for example, phasic inhibition in the basolateral amygdala (BLA) is reduced in *GAD65* knockout mice (−/−) [16]. In addition, Mice exposed to prenatal stress developed an anxiety-like phenotype, which was accompanied by a substantial reduction in *GAD67* expression in the BLA [21]. Moreover, GAD expression in the amygdala was shown to be reduced by stress, both at the mRNA and protein levels in mice [14]. In line with these results, Stone et al., demonstrated in 2001 that corticosterone (CORT) treatment altered GAD genes expression in the dentate gyrus (DG) and the CA1 region of the hippocampal circuit [19]. Another research group demonstrated that rats exposed to one hour of stress in plastic restraint tubes had an increase in *GAD67* mRNA expression in the CA1 and DG and increased *GAD65* expression only in the DG [22]. Hadad-Ophir et al. (2014) revealed reduced expression levels of *GAD65* and *GAD67* in rats 5 h after spatial learning in a Morris water maze (MWM). These expression patterns were also compared to changes in a different group of rats, subjected to the same water maze but without access to an unseen escape platform; in that group, there was a lesser expression reduction that was observed in the dorsal CA1 [15]. In addition, it was found that mRNA levels of *GAD65* in the dorsal CA1 were higher in rats after exposure to either juvenile stress (JS) or adult stress (AS) in comparison to rats exposed to both types of stress [23]. Since the behavioral phenotype of rats subjected to combined JS + AS is more severe than in rats exposed to a single type of stress [23], Albrecht et al., 2017, suggested that such an expression pattern may be related to stress resilience. Thus, increased *GAD65* expression may contribute to the stress systems’ attempts to restabilize, a process that is impeded by the combination of JS + AS. Interestingly, stress-induced neurobiological alterations may contribute to vulnerability but also to potential resilience to stress experiences in adulthood [23].

Epigenetics is the study of heritable gene function changes at the mitotic or meiotic level that do not involve changes in the DNA sequence itself. Various epigenetic mechanisms can influence gene expression through gene amplification and silencing [24]. External or environmental factors can cause epigenetic changes with cellular and physiological phenotypes [25]. One of the mechanisms by which epigenetics can regulate gene expression is through DNA methylation, a covalent DNA modification (activated by DNA methyltransferase (DNMT, DNA MTase) enzyme family) that places a methyl group on cytosine nucleotides at the c5 position [26]. Methylation of genomic DNA has primarily been observed in the context of cytosine methylation in CpG sites and is regarded as the most common epigenetic modification [27]. CpG sites are locations in the DNA molecule’s linear sequence where a cytosine nucleotide occurs first, followed by a guanine nucleotide. As one of the most stable components of the epigenome, DNA methylation is responsible for the establishment and stabilization of cell-specific phenotypes via gene expression level maintenance [28].

Considerable evidence shows that exposure to stress can cause long-lasting epigenetic changes that lead to gene expression alterations and affect the whole organism [29]. In many aspects of adult human lives, during periods of chronic stress, such as combat exposure [30], employment in a high-stress environment [31], or a high lifetime stress load [32], epigenetic alterations can be found. Epigenetic alterations may also be involved in the lasting impact of childhood trauma. Preclinical studies have shown a consistent link between the early life environment, DNA methylation changes, and adult stress reactivity and behavior [33,34]. For example, a study of rodents exposed to stress early in life, such as impaired maternal behavior, has shown long-lasting epigenetic alteration of stress-associated genes [34,35]. Similar epigenetic changes were observed in humans exposed to childhood maltreatment, such as the development of mood disorders or PTSD later in life [36]. The long-term impact of traumatic stress on DNA methylation patterns is supported by several studies, which mainly focused on single genes [37]. Thus, stress and trauma are environmental exposures that can cause lasting epigenetic changes and lead to the development of psychiatric pathology.

The promoter region of genes has received the most attention since it is generally associated with the repression of transcription [38,39]. Recent studies suggest that DNA methylation in other locations, both upstream and downstream of the transcription start sites, has an equally essential role of transcription regulation [40,41,42]. In addition, Anastasiadi et al. (2018) presented an inverse correlation of the DNA methylation in the first intron and gene expression [43]. Additionally, only a few studies investigated or found results regarding the methylation of the first intron of GAD genes, specifically as a long-term effect.

Here, we hypothesized that DNA methylation might affect *GAD*’s isoforms gene expression in the hippocampus and the amygdala. We tested whether methylation changes and mRNA expression will vary between three rat groups: a. rats exposed to juvenile stress, b. rats exposed to both juvenile and adult stress, and c. a control group of rats that were not exposed to any stress during their lifetime. The findings of this study may shed some light on the mechanism by which those alterations occurred, and they may also suggest a new strategy for a specific intervention in future research and clinical practice.

## 2. Results

### 2.1. Behavioral Tests

**Figure 1:**
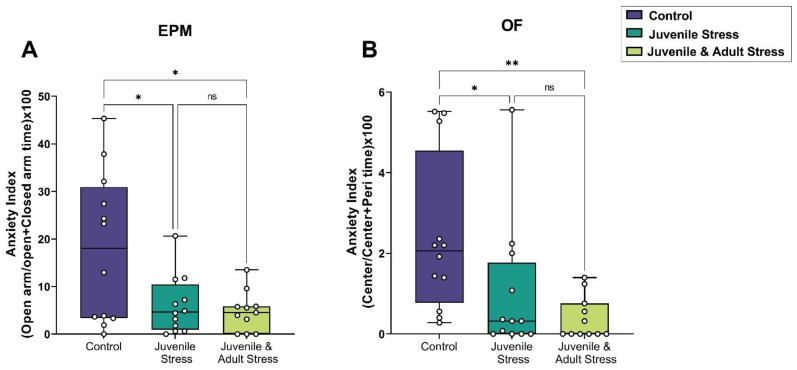
Impact of juvenile stress on anxiety behavior as measured by the elevated plus maze and open field tests. (**A**) Anxiety index in elevated plus maze (EPM) test shows stress-induced anxiety in juvenile stress (JS) group and JS and adult stress (JS + AS) group compared to the control group, statistically defined by Kruskal–Wallis main group effect, *p* < 0.01, a lower anxiety index among JS and JS + AS groups compared to control, *p* < 0.05 (**B**) anxiety index of open field (OF) shows significant one-way ANOVA main group effect, *p* < 0.01, a lower anxiety index (meaning higher anxiety behavior) among JS compared to control, *p* < 0.05, and JS + AS compared to control, *p* < 0.01. All figures are depicted with mean ± SEM of 12 (control), 12 (JS), and 11 (JS + AS). * *p* < 0.05 ** *p* < 0.01.

Anxiety indices were calculated for each test (open field (OF) and elevated plus maze (EPM)) in order to validate the behavioral phenotype (a lower anxiety index points to higher anxious behavior). As expected, one-way ANOVA revealed significant differences between the groups (*p* < 0.01): a lower anxiety index was shown among JS and JS + AS groups compared to control (*p* < 0.05 for both). Similarly, an OF test also revealed significant differences among groups (*p* < 0.01) with the same pattern as EPM: JS and JS + AS groups showed a lower anxiety index compared to the control group (*p* < 0.05, *p* < 0.01 respectively), using Kruskal–Wallis and Dunn’s tests, hence, exhibited more anxious behavior (Figure 1).

### 2.2. Sequenom Epityper MassARRAY

Epiloci methylation level differences of the *GAD65* gene (52 CpG sites in total) and its proximity were assessed in three independent groups: control, juvenile stress (JS), and juvenile and adult stress (JS + AS). Methylation patterns of the stress-related candidate gene were tested at each brain region (dorsal dentate gyrus (dDG), dorsal CA1 (dCA1), and the basolateral amygdala (BLA)). For statistical analysis, we used the Kruskal–Wallis non-parametric test to find differences in methylation percentage between the three groups for each CpG site. Additional pairwise multiple comparisons were conducted to evaluate each group’s contribution.

Table 1 presents the means and standard error (SE) of methylation percentage of the explored gene in each brain region for each group, in addition to the adjusted significance for significant comparison. Comparisons of the three groups in three different brain regions regarding gene first intron methylation patterns revealed significant trends.

**Figure 2:**
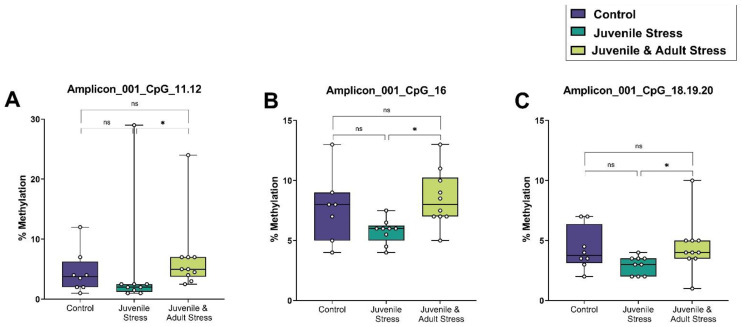
Differential methylation in three CpG site of *GAD65* in the BLA among juvenile stress, juvenile and adult stress, and control groups. (**A**) Amplicon_001_CpG_11.12, significantly lower methylation among JS compared to JS + AS, *p* < 0.05, control *n* = 8, JS *n* = 9, JS + AS *n* = 10; (**B**) Amplicon_001_CpG_16, significantly lower methylation among JS compared to JS + AS, *p* < 0.05, control *n* = 7, JS *n* = 9, JS + AS *n* = 10; (**C**) Amplicon_001_CpG_18.19.20, significantly lower methylation among JS compared to JS + AS, *p* < 0.05, control *n* = 8, JS *n* = 9, JS + AS *n* = 10. * *p* < 0.05, ns (non-significance).

Two CpG sites in the *GAD65* methylation pattern in the BLA were found to be statistically significant between the tested groups (amplicon_001_CpG_11.12: *p* < 0.05, amplicon_001_CpG_16: *p* < 0.05). A pairwise comparison resulted in a significant difference between the JS + AS and the JS groups for the three sites (Table 1), where the JS + AS group had slightly more methylation than JS (Figure 2).

**Figure 3:**
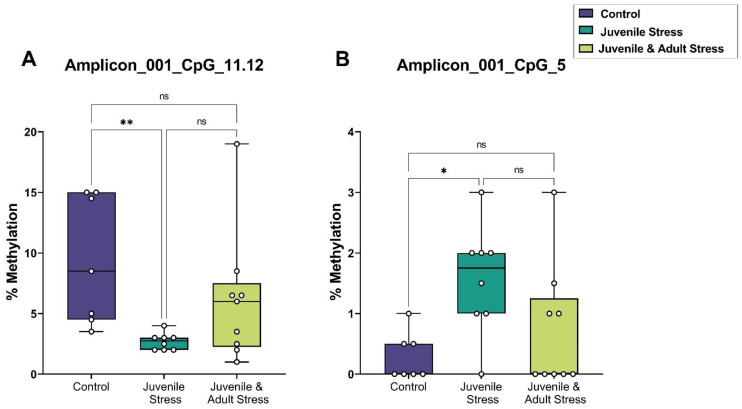
Differential methylation in two CpG sites of *GAD65* in the dCA1 among juvenile stress, juvenile and adult stress, and control groups. (**A**) Amplicon_001_CpG_11.12, significantly lower methylation among JS compared to control, *p* < 0.01, (**B**) Amplicon_001_CpG_5, significantly higher methylation among JS compared to control, *p* < 0.05, control *n* = 7, JS *n* = 8, JS + AS *n* = 9. * *p* < 0.05 ** *p* < 0.01, ns (non-significance).

**Figure 4:**
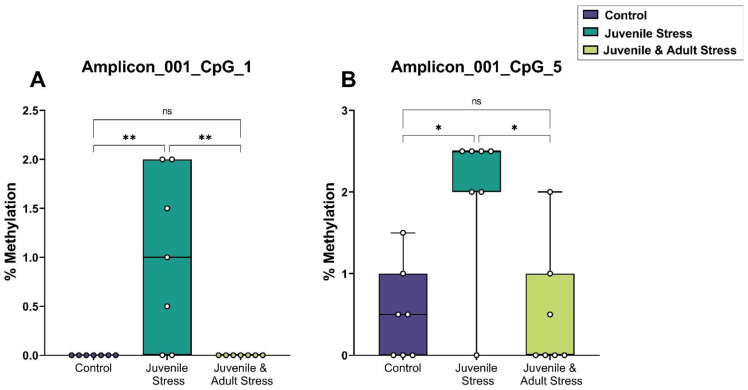
Differential methylation in two CpG sites of *GAD65* in the dDG among juvenile stress, adult stress, and control groups. (**A**) Amplicon_001_CpG_1, significantly lower methylation among control and JS + AS groups compared to JS, *p* < 0.01 for both, (**B**) Amplicon_001_CpG_5, significantly higher methylation among JS compared to control and JS + AS groups, *p* < 0.05 for both, control *n* = 7, JS *n* = 7, JS + AS *n* = 7. * *p* < 0.05 ** *p* < 0.01, ns (non-significance).

In the dCA1, two CpG sites were found to be significant compared to the three groups. Amplicon_001_CpG_11.12 (*p* < 0.05) was also found to be significant in BLA; in this site, pairwise comparisons revealed significant differences between the JS group and the control, in favor of the latter. Amplicon_001_CpG_5 in this region was also found to be statistically significant (*p* < 0.05), but, here, JS group had more methylation than the control group (Table 1, Figure 3). Changes in the dDG region methylation were also revealed to be statistically significant in amplicon_001_CpG_5 site (*p* < 0.05) and amplicon_001_CpG_1 site (*p* < 0.05). The JS group had significantly higher methylation in both sites than the JS + AS and control groups (Figure 4).

**Figure 5:**
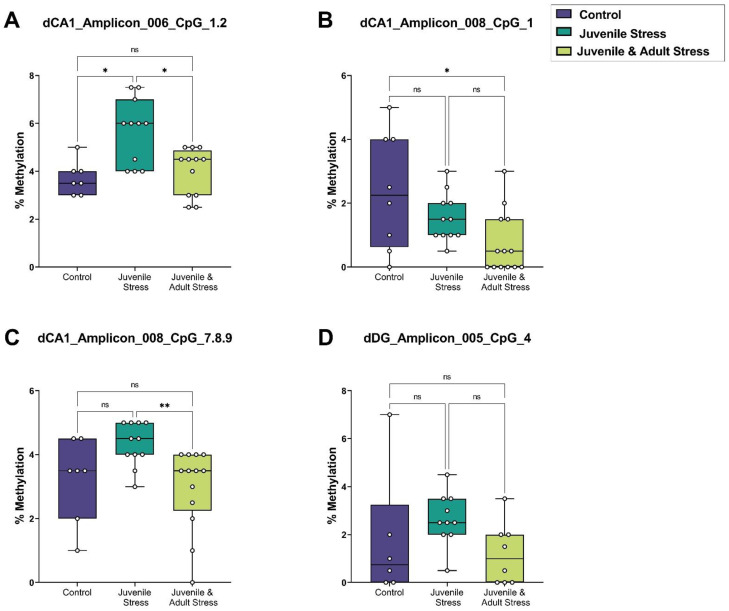
Differential methylation of three CpG sites of *GAD67* in the dCA1 and one CpG site of *GAD67* in the dDG and (**A**) dCA1_Amplicon_006_CpG_1.2, significantly lower methylation among control and JS + AS groups compared to JS, *p* < 0.05 for both, control *n* = 7, JS *n* = 11, JS + AS *n* = 12, (**B**) dCA1_Amplicon_008_CpG_1, significantly higher methylation among control compared to JS + AS groups, *p* < 0.05, control *n* = 8, JS *n* = 11, JS + AS *n* = 13. (**C**) dCA1_Amplicon_008_CpG_7.8.9, significantly higher methylation among JS compared to JS + AS group, *p* < 0.01, control *n* = 7, JS *n* = 11, JS + AS *n* = 13. (**D**) dDG_Amplicon_005_CpG_4, trend toward significantly higher methylation among JS compared to JS + AS groups, *p* = 0.06, control *n* = 6, JS *n* = 10, JS + AS *n* = 8. * *p* < 0.05 ** *p* < 0.01, ns (non-significance).

Methylation percentage patterns of *GAD67* in the brain regions demonstrated significant differences as well. In the dDCA1 region, three CpG sites showed significant differences between the groups: amplicon_006_CpG_1.2, amplicon_008_CpG_1, and amplicon_008_CpG_7.8.9 (*p* < 0.05); each site showed a different pattern of differences between the groups in the analysis of the pairwise comparison. In amplicon_006_CpG_1.2, the JS group had a significantly higher methylation percentage than the control group and the JS + AS group. In amplicon_008_CpG_1, the control group showed more methylation than the JS + AS group. In amplicon_008_CpG_7.8.9, the JS group showed more methylation than the JS + AS group. Significant differences between the groups were discovered in the dDG in amplicon_005_CpG_4 (*p* < 0.05), while pairwise comparison revealed only a trend toward significance. The JS + AS group showed a slightly lower methylation percentage compared to JS. (Table S1, Figure 5).

**Figure 6:**
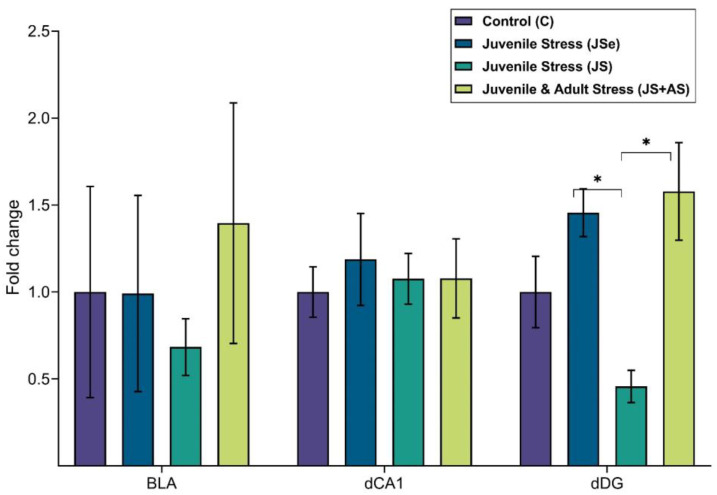
Differences in *GAD65* mRNA expression across the treated groups in the dDG, dCA1, and BLA. The JS group showed significantly lower mRNA expression compared to the JS + AS and control groups, as indicated by Kruskal–Wallis results (*p* < 0.01 for both comparisons). Control *n* = 4, JSe *n* = 5, JS *n* = 5, JS + AS *n* = 4. * *p* < 0.05.

**Figure 7:**
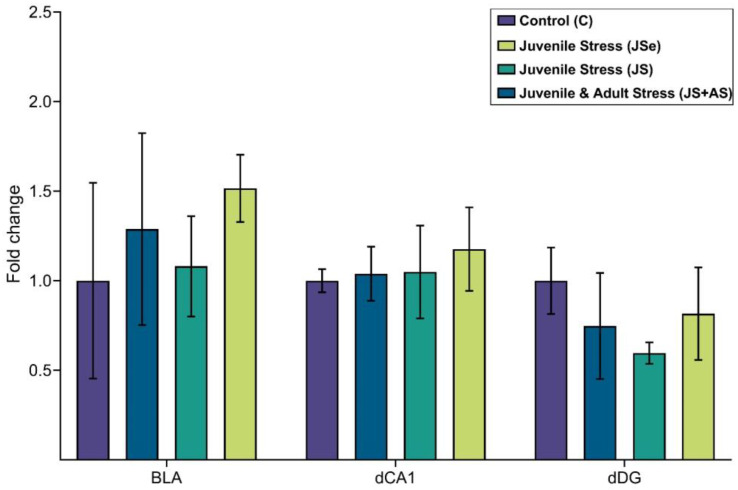
Comparison of *GAD67* mRNA expression levels among the different groups in the dDG, dCA1, and BLA brain regions. No significant differences in expression were observed between the groups. Control *n* = 4, JSe *n* = 5, JS *n* = 5, JS + AS *n* = 4.

We also examined the mRNA patterns in a different group of rats, in which the rats were sacrificed two days following juvenile stress (JSe). Among two genes (*GAD65* and *GAD67*), three brain regions (BLA, dCA1, and dDG), and between the four groups (control, JS, JS + AS, and JSe), a significant difference was found in dDG *GAD65* RNA expression (*p* < 0.05). *GAD65* expression was higher in JS + AS compared to the JS group, while the expression in the JS group was significantly lower compared to the JSe. Although not significant, a lower *GAD65* expression was found for the JS group compared to the control (Figure 6). No differences were found in *GAD67* mRNA (Figure 7).

## 3. Discussion

The results of the behavioral tests in this study demonstrate that exposure to both juvenile and adult stress or only juvenile stress has a significant impact on stress levels compared to the control group. Furthermore, numerous studies have established the association between juvenile exposure to stress and stress reactions in adulthood. In adulthood, genetic and environmental factors may contribute to the onset of a stress response. Thus, we aimed to delve deeper into the molecular mechanisms underlying this effect. Here, we tested whether DNA methylation could act as a biomarker for predicting stress response. We showed that the first intron methylation of the *GAD65* gene in dDG and BLA can serve as such a biomarker, as it is differentially methylated among the three groups in the study.

In dDG, two CpG sites in the first intron of *GAD65*, between the first and second exons, produced the most notable results. The methylation percentage was significantly higher among rats that were exposed to both types of stress (juvenile and adult) compared to rats exposed to juvenile stress alone. Methylation in the first intron was shown to be negatively correlated with gene expression [43]. In accordance, we observed lower gene expression levels among the JS group compared to both the control group and the JS + AS group. Stone et al. (2001) reported a similar result, in which *GAD65* expression decreases following a single exposure to stress hormone and increases following two exposures [19]. Further, Müller et al. (2014) demonstrated that *GAD65* haplodeficiency (*GAD65*+/−) confers stress-resilience in animals with JS history [44]. Tripathi et al. (2021) demonstrated the effects of a reduction in *GAD65* expression. Knocking down *GAD65* in the dDG of animals as adults significantly reduced their innate anxiety level. In animals exposed to JS, reduced *GAD65* expression in the dDG appears to protect against JS-induced anxiety and cognitive impairment [45]. It is possible that the sharp rise in *GAD65* RNA in dDG immediately following a stress event, as demonstrated by our findings and supported by previous research [22,46], necessitates adaptations that result in increased methylation as a long-term effect. *GAD65*′s short-term effects on stress are well established. As mentioned earlier, while the hippocampus sends inhibitory signals via glutamatergic projections in GABAergic PVN-projecting neurons, the amygdala excites the PVN via disinhibition signals [3,4]. Chronic stress has been shown to increase the availability of *GAD65* in the amygdala [22]. While previous studies showed the short-term effect of *GAD65* following stress, in this study, we offer evidence of a long-term effect. It has been shown that animals exposed to juvenile stress had higher expression of GABAA receptor subunits in the BLA if exposed to stressors later in life. In the same study, a small trend toward lower GABAA subunits’ expression was found in animals exposed to JS solely compared to the control group of animals [47]. Along these lines, while there were no differences in *GAD65* expression in the BLA, we did observe that rats exposed to juvenile stress alone had a significantly lower DNA methylation percentage in three CpG sites on the first intron of *GAD65* when compared to rats exposed to both juvenile and adult stress or no stress at all. This lower methylation level can be interpreted as the system’s long-term attempt to cope with the severe traumatic event during juvenility.

Another interesting aspect of these findings is the methylation pattern. As can be seen from the methylation distribution patterns, the JS + AS and control groups exhibit similar methylation patterns, whereas the JS demonstrates a distinct pattern. This similarity between the JS + AS and control groups may reflect recovery due to adulthood stress, “fixing” the symptoms caused by juvenile stress. Moreover, Albrecht et al. (2017) proposed that coping with multiple stressors throughout life could “train” the subject. Repeated stressful events cause neurobiological changes that may contribute to vulnerability, yet also to potential resilience to stress experiences in adulthood and thus help the subject cope with stress more effectively [23]. We suggest the possibility that this “training” modifies DNA methylation in order to rehabilitate the stress system. Ardi et al. (2016) demonstrated that the proportion of animals severely affected by stressor exposure (as determined by behavioral tests) was significantly higher in a group of animals exposed to both types of stress (JS + AS) than in a group of animals exposed to JS alone, but, importantly, even under these conditions, a significant proportion of the exposed animals did not develop pathology [48]. This is in line with findings in humans, indicating that the majority of individuals exposed to a traumatic experience do not develop pathology [49].

Another possibility is that, while the control and JS + AS groups have similar GAD methylation patterns, they differ in other gene expression and methylation patterns, implying that different mechanisms underlie the molecular or biochemical response to stress, i.e., various changes in gene expression occur as a result of additional epigenetic modifications mediated by other mechanisms involved in the response seen in the JS + AS group. For example, we hypothesize that genes related to the endocrine system may play a role. Cortisol is a major factor in stress response that contributes to the system coping with threatening situations. It is well known that exposure to stress induces the release of cortisol [50]. Evidently, childhood trauma is associated with alterations of the HPA axis in the pituitary, which releases cortisol [51,52]. In addition, exposure to trauma early in life is a major risk factor for developing psychiatric disorders in general and specifically PTSD [53,54].

**Scheme 1:**
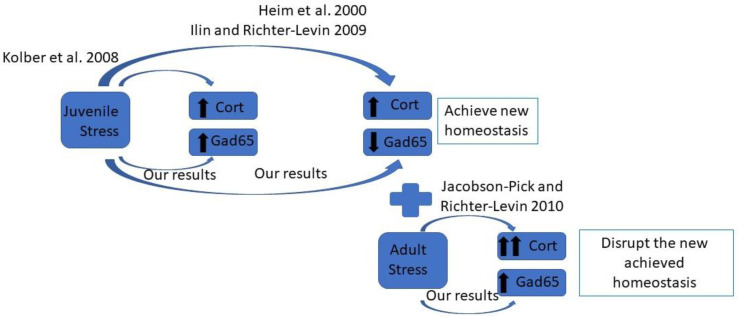
Schematic representation of potential homeostatic disruptions caused by juvenile stress alone and the addition of adult stress [50,52,59,60].

It can be suggested that exposure to stress early in life disrupts homeostasis and affects the endocrine system in a variety of ways, including initial changes in cortisol release and demand, as well as changes in the methylation and expression of *GAD65* to cope with it. In adulthood, the second exposure to stress alters the methylation patterns of *GAD65* again, resulting in increased expression of *GAD65* in an attempt to handle cortisol levels. Those changes can lead to the development of anxiety disorders and non-adaptive behaviors (Scheme 1).

Moreover, human studies confirmed that childhood trauma affects cortisol levels via epigenetic mechanisms [55,56]. Accordingly, juvenile stress in rodents induces a disturbance of basal corticosterone plasma levels and stress-induced corticosterone secretion that can last even into adulthood [57,58,59]. Specifically to our stress model, it was found by llin and Richter-Levin (2009) that exposure to the same three days model of stress during juvenility affected the HPA axis baseline activity. Analysis of basal circulating CORT levels revealed long-lasting elevated levels in the JS group compared to the control group [60], while additional stress in adulthood markedly exceeded the corticosterone compared to juvenile stress alone or to non-stressed animals.

Although we believe that this time frame is appropriate for our study design, it is possible that other methylation patterns may emerge if the JS + AS group was examined after a longer time period (such as 45 days, as was conducted with the JS group). Additionally, it should be noted that differences in procedure between the JS and JS + AS groups may affect the interpretation of the findings, as the JS group underwent a different procedure before being sacrificed. Another limitation is the inability to analyze the correlation between RNA and methylation percentage, as samples were taken from two different animal groups. Further studies will be needed to address this gap in the current research and to establish the relationship between these two variables. It is also worth noting that the prefrontal cortex is known to play a significant role in the stress response [61] (in addition to the hippocampus and amygdala), and methylation in this area of the brain should be studied in future research.

## 4. Methods

### 4.1. Animals

Male Sprague–Dawley rats (postnatal day (PND) 22 on arrival, 30–50 g, (Harlan Laboratories, Jerusalem, Israel)) were group housed, four per cage (22 ± 2 °C; light–dark cycle: 12/12 h), with ad libitum water and food. All experiments were performed according to the NIH Guide for care and use of laboratory animals and approved by the University of Haifa Ethical Committee (430/16).

### 4.2. Experimental Groups

#### 4.2.1. Methylation Analysis

Following a five-day post-delivery acclimation period, animals were randomly assigned to one of three groups: (1) control (*n* = 15), (2) juvenile stress ((JS), *n* =15), or (3) adult stress (underwater trauma (UWT) (AS), *n* = 15).

#### 4.2.2. Expression Analysis

Another group of animals was randomly assigned to one of four groups: (1) control (*n* = 4), (2) juvenile stress early sacrificed ((JSe) (*n* = 5)), (3) JS (*n* = 5), and (4) JS + AS (*n* = 4). The control rats were left undisturbed in their home cage until PND 75. At PND 27–29, JS rats were subjected to “juvenile stress model” protocols; while the JSe group was sacrificed two days later, the JS group was sacrificed at PND 60. JS + AS rats were also produced by subjecting the animals to UWT (PND 60) and then sacrificing them at 75 PND.

### 4.3. Stress Protocols and Behavioral Assessments

In the present study, a well-established rat model of PTSD was utilized, combining juvenile stress (JVS) exposure and subsequent adult exposure to underwater trauma (UWT) [62]. JVS exposure consisted of three different stressors applied over three consecutive days (PND 27–29) under full light conditions, as described before [63]. The UWT procedure (PND 60) was conducted following the method described by [64]. After two weeks of UWT, rats were tested on the open field (OF) and elevated plus maze (EPM) to evaluate the impact of the stress protocol. The OF test was performed at PND 73, and the EPM test was carried out after 24 hours [65,66]. The behavior of the animals during the OF and EPM tests was recorded and analyzed using the EthoVision XT8 video tracking system (Noldus, Wageningen, Netherlands). The anxiety index for the OF test was calculated by dividing the time spent in the center by the total time spent in the center and periphery, multiplied by 100. The anxiety index for the EPM test was calculated as the ratio of time spent in the open arm to the total time spent in both the open and closed arms, multiplied by 100. Lower anxiety indices indicate higher anxiety levels.

### 4.4. Harvesting Brain Tissues

Rats were sacrificed at PND 75, one day following the last behavioral test. Brains were taken out for biochemical analysis from all rat groups. Brains were snap-frozen with cryostat apparatus (at −20 °C; Leica, Wetzlar, Germany); frozen tissue punches (diameter: 1 mm; depth: 1.5 mm) were collected from each hemisphere covering the rostro-caudal axis of the basolateral amygdala complex (BLA; −1.8 mm from Bregma), the dorsal dentate gyrus (dDG), and the dorsal cornu ammonis 1 (dCA1; both starting −2.8 mm from Bregma), according to the Paxinos and Watson brain atlas [67].

**Scheme 2:**
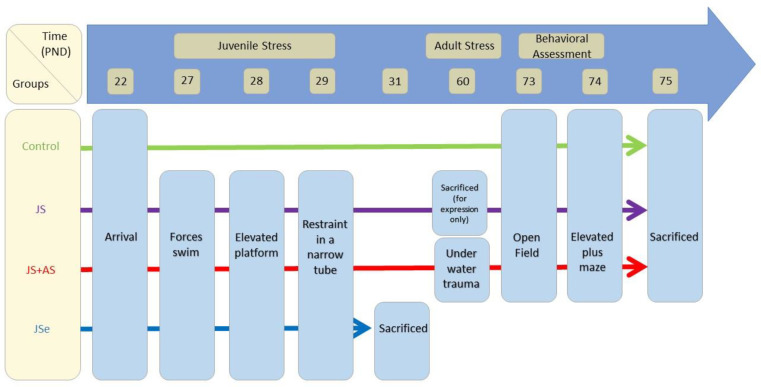
Experimental timeline. Each experimental group undergoes conditioning as shown. CTR = Control, JS = Juvenile stress, JS + AS = Juvenile and adult stress.

All rat treatments, stress exposures, and behavioral tests are presented in Scheme 2.

### 4.5. DNA Extraction

DNA samples were extracted from animals’ tissues using the High Pure PCR Template Preparation Kit (Roche, Penzberg, Germany) according to the manufacturer’s instructions.

### 4.6. Bisulfite Treatment

DNA was subjected to bisulfite treatment using the EZ DNA Methylation-Lightning™ Kit (ZYMO RESEARCH, Irvine, CA, USA). Briefly, the conversion reaction was performed in a 150 μL mixture containing 20 μL of genomic DNA (about 500 ng) and 130 μL of bisulfite solution. After conversion, DNA was washed according to the protocol and eluted with 20 μL elution buffer.

### 4.7. Amplicon and Primer Selection

**Figure 8:**
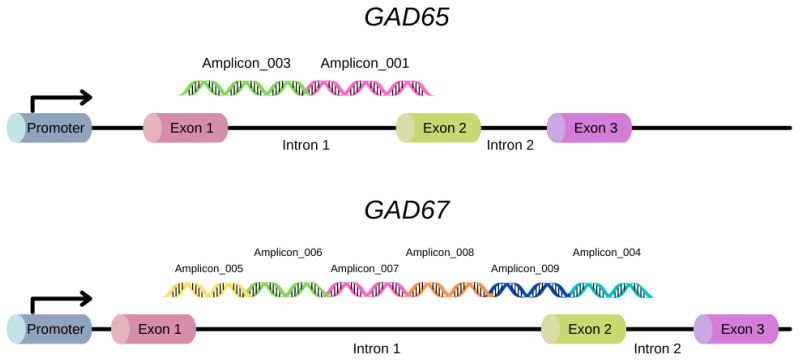
Location of designed primers’ amplicons for each gene (created with Canva).

The CpG-rich sequences for *GAD65* were selected using the NCBI genome browser (https://www.ncbi.nlm.nih.gov/genome (accessed on 9 July 2019)) and CpG Island finder browser (http://dbcat.cgm.ntu.edu.tw/ (accessed on 9 July 2019)) URLs (accessed on 9 July 2019). Primers were designed using the Sequenom EpiDesigner software (http://www.epidesigner.com (accessed on 9 July 2019)), taking into account amplicon coverage, number of CpGs, fragment size, and the number of nucleotide repeats in the primer sequence (The characteristics of amplicons are reported in Table S2, and the sequences of the primers are reported in Table S3). Figure 8 depicts the primers’ coverage of gene locations.

### 4.8. Polymerase Chain Reaction (PCR) Amplification

PCR amplification reactions for each amplicon were designed to contain 10 ng of bisulfite-treated DNA. All other PCR reagents were added as described in the EpiTYPER protocol.

### 4.9. Preparation for Sequenom

Following DNA amplification, a shrimp alkaline phosphatase treatment was performed. PCR products (2 μL) were used as a template for in vitro transcription and RNAse A cleavage for the T-reverse reaction as described in the manufacturer’s instructions (Sequenom hMC).

### 4.10. Sequenom Epityper MassARRAY Methylation and Spectra Analysis

Samples were desalted and spotted on a 384-alternative patch SpectroCHIP (Sequenom, San Diego, CA, USA) using the MassARRAY nanodispenser, and spectra were analyzed by the MassARRAY Analyzer Compact matrix-assisted laser desorption/ionization–time of flight (MALDI-TOF MS) (Sequenom, San Diego, CA, USA). Spectra were elaborated by the Epityper 1.2 software (Sequenom, San Diego, CA, USA), which provides methylation values for each CpG unit as a percentage. Each CpG unit is determined by the EpiTYPER software and can be represented by one or more CpG sites. Methylation percentages for each CpG unit are calculated as a mass signals ratio between methylated and non-methylated DNA based on the elaboration of fragment masses generated during RNase A-specific cleavage.

### 4.11. RNA Extraction and cDNA Synthesize

mRNA was extracted from the different brain regions previously described using TRIzol™ (Invitrogen, Carlsbad, CA, USA) according to the manufacturer’s instructions. Briefly, dissected brain regions were homogenized in 1000 µL of TRIzol Reagent and one μL glycogen (Sigma-Aldrich, Schnelldorf, Germany) and centrifuged at 12,000× *g* for 5 min, 4 °C. The clear supernatant was transferred to a new tube and incubated for 5 min at room temperature. An amount of 200 µL of chloroform was added, and samples were incubated for 2–3 min and again centrifuged at 12,000× *g* for 15 min, 4 °C. The aqueous phase containing the RNA was transferred to a new tube, and 500 µL of isopropanol were added, incubated for 10 min, and centrifuged again at 12,000× *g* for 10 min, 4 °C. The pellet was washed with 1000 μL cold 75% ethanol, centrifuged at 7500× *g* for 5 minutes, and dried. RNA was diluted in 40 µL RNase-free water, and quantities were determined using a Nanodrop 2000 spectrophotometer (Thermo Scientific, Waltham, MA, USA).

Single-stranded cDNA was synthesized using iScript™ cDNA Synthesis Kit (BioRad, Hercules, CA, USA) according to the manufacturer’s instructions. Shortly, a total of 500 ng was added to four µL of 5X iScript Reaction Mix and one µL iScript Reverse Transcriptase. Synthetic cDNA was amplified using specific primers (Table S4).

### 4.12. Quantitative RT-PCR

cDNA was amplified using Fast SYBR™ Green Master Mix (Applied Biosystems™, Vilnius, Lithuania). The PCR amplification was performed using the LightCycler 480 II (Roche, Penzberg, Germany). Fold-change values were calculated using the ΔΔCt method relative to the housekeeping gene (HKG) hypoxanthine phosphoribosyl transferase (Hprt), which is commonly used as a reference gene.

### 4.13. Statistical Analysis

For behavioral measurement, each data set was tested for normality distribution and equal variance by Shapiro–Wilk test. According to the normality results, EPM anxiety indices were tested using ordinary one-way ANOVA followed by Tukey’s multiple comparisons test. The anxiety index of OP did not exhibit a normal distribution of values, hence tested using Kruskal–Wallis followed by Dunn’s multiple comparisons test.

The average methylation percentage for each gene and its proximate region was calculated for all samples. The Kruskal–Wallis rank-sum test was conducted to compare methylation distributions across all groups. Dunn’s multiple comparisons test was performed, comparing the gene methylation means of each group to the same variable measured in the other groups. To find significant differences in mRNA expression among the four groups, the Kruskal–Wallis rank-sum test and Dunn’s as post hoc test was used. SPSS software version 25 (IBM) was used to analyze the data (SPSS Inc., Chicago, IL, USA).

## Figures and Tables

**Table 1:** Mean methylation percentages and SE of the candidate GAD65 epigenetic loci in each brain region among the three groups studied.

Region	CpG Site	Control			JS			JS + AS			Adj. Sig.		
		*n*	M%	SE%	*n*	M%	SE%	*n*	M%	SE%	JS vs. Control	JS + AS vs. Control	JS + AS vs. JS
BLA	amplicon_001_CpG_11.12	8	4.4	1.2	9	4.9	3	10	6.9	2	0.652	0.439	0.015 *
	amplicon_001_CpG_16	7	7.7	1.1	9	5.8	0.3	10	8.5	0.7	0.231	1	0.019 *
	amplicon_001_CpG_18.19.20	8	5.3	0.6	9	3	0.3	10	4.5	0.7	0.216	1	0.044 *
dCA1	amplicon_001_CpG_11.12	7	9.4	2	8	2.7	0.2	9	6.2	1.8	0.009 **	0.477	0.258
	amplicon_001_CpG_5	7	0.3	0.1	8	1.6	0.3	9	0.7	0.3	0.031 *	1	0.144
dDG	amplicon_001_CpG_1	7	0	0	7	1	0.3	7	0	0	0.007 **	1	0.007 **
	amplicon_001_CpG_5	7	0.5	0.2	7	2	0.3	7	0.5	0.2	0.045 *	1	0.029 *

* *p* < 0.05, ** *p* < 0.01.

## Data Availability

Data is contained within the article or supplementary material.

## Supplementary Materials

The following supporting information can be downloaded at: https://www.mdpi.com/article/10.3390/genes14030565/s1, Table S1: Means methylation percentage and SE of the candidate GAD67 epigenes loci in each brain region among the three studied groups; Table S2: The characteristics of all CpG islands amplicons in GAD65 and GAD67 genes; Table S3: Primers sequences; Table S4: Primers sequences for RT-PCR.
