# Supplementary material for: Exposure to Juvenile Stress Induces Epigenetic Alterations in the GABAergic System in Rats
Source: Genes (Basel). 2023 Feb 23;14(3):565. doi: 10.3390/genes14030565 (PMC10048220; doi:10.3390/genes14030565)
Supplement: Supplementary file 1 [file genes-14-00565-s001.zip › genes-2188068-supplementary.pdf]

Supplemental data

Table S1: Means methylation percentage and SE of the candidate *GAD67* epigenes loci in each brain region among the three studied groups.

Region	CpG Site	Control			JS			JS+AS			Adj. sig.		
		N	M%	SE%	n	M %	SE %	n	M%	SE %	JS vs. Control	JS+AS vs. Control	JS+AS vs. JS
**dCA1**	amplicon_006_CpG_1.2	**7**	**3.7**	**0.3**	**11**	**5.7**	**0.4**	**12**	**4**	**0.3**	**0.012***	**1**	**0.045***
	amplicon_008_CpG_1	**8**	**2.4**	**0.6**	**11**	**1.5**	**0.2**	**13**	**0.7**	**0.3**	**1**	**0.039***	**0.104**
	amplicon_008_CpG_7.8.9	**7**	**3.2**	**0.5**	**11**	**4.3**	**0.2**	**13**	**3**	**0.3**	**0.112**	**1**	**0.008****
**dDG**	amplicon_005_CpG_4	**6**	**1.7**	**1**	**10**	**2.6**	**0.3**	**8**	**1.2**	**0.4**	**0.161**	**1**	**0.062**

*p<0.05 **p<0.01 ***p<0.001

Table S2: The characteristics of all CpG islands amplicons in *GAD65* and *GAD67* genes

Gene	Chr.	Position	Primer No.	Specific region start-end position	Product size	CpG sites
GAD67	3	56861261-3238	2	157-545	389	25
			5	521-799	279	26
			6	768-1173	406	17
			11	1136-1399	264	11
			14	1360-1777	418	29
			16	1751-1909	159	8
GAD65	17	89171099-2875	1	562-948	387	13
			8	924-1338	415	30
			14	1314-1696	383	32

Table S3: Primers sequences. R-Reverse Primer Plus T7 Tag, F- Forward Primer Plus 10 mer Tag

Primer name		Target Length
GAD67-2	R-5'-cagtaatacgactcactatagggagaaggctATCACTTCAACCCCTATATATCCTC-3'<br>F-5'- aggaagagagGGATTAGGGATTTTGTAAGTAAGGAA-3'	389
GAD67-5	R-5'-cagtaatacgactcactatagggagaaggctAAAAACACTAATCTAAATAAATCTAAAACC-3'<br>F-5'-aggaagagagGAGGATATATAGGGGTTGAAGTGAT-3'	279
GAD67-6	R-5'-cagtaatacgactcactatagggagaaggctAAACCAATCACCTTACACTCCAAAC-3'	406

	F-5'aggaagagagGGGGTTTTAGATTTATTTAGATTAGTG-3'	
GAD67-11	R-5'-cagtaatacgactcactatagggagaaggctCCAACTAAAACAAACCCCATTAAA-3'<br>F-5'aggaagagagTAGTTTTTGGTTGTTTGGAGTGTA-3'	264
GAD67-14	R-5'-cagtaatacgactcactatagggagaaggctAAAATCTCTACTATTCAAATATCCCCC-3'<br>F-5'aggaagagagGTTAGGTTGTTGGGAGTTTAATGG-3'	418
GAD67-16	R-5'-cagtaatacgactcactatagggagaaggctCTACAATCCATTTAATAAAAAACTAAAACA-3'<br>F-5'aggaagagagGGGGGATATTTGAATAGTAGAGATTTT-3'	159
GAD65-1	R-5'-cagtaatacgactcactatagggagaaggctATTCCTAAATTACCTTTCCCTACCC-3'<br>aggaagagagGGGTTTTGGTTTTTGGTTTTT-3'	387
GAD65-8	R-5'-cagtaatacgactcactatagggagaaggctTTAAACCACCTAACACCAAACTCTC-3'<br>F-5'aggaagagagGGGTAGGGAAAGGTAATTTAGGAAT-3'	415
GAD65-14	R-5'-cagtaatacgactcactatagggagaaggctACATAATTAACATCTCCTTTAAAACAA-3'<br>F-5'aggaagagagGAGAGTTTGGTGTTAGGTGGTT-3'	383

Table S4 : Primers sequences for RT-PCR

Gene name	RT-PCR Forword primer	RT-PCR Reverse primers
Gad67	5′CCCAAACTGGTCCTCTTCAC3′	5′ TGTCAGTTCCAAAGCCAAGC3′
Gad65	5′AGGTGGCCCAAAAGTTCAC3′	5′TTGCGTGGAGAAGTGCATAA3′
HPRT	5′CGCCAGCTTCCTCCTCAG3′	5′ATAACCTGGTTCATCATCACTAATCAC3′
